# Bedside Sonography Brings the Weiss Ring into Focus: Rapid Diagnosis of Posterior Vitreous Detachment

**DOI:** 10.5811/cpcem.63028

**Published:** 2026-10-13

**Authors:** Joseph Betcher, Matthew Shields, Dakota Busch

**Affiliations:** Michigan State University, Trinity Health Muskegon, West Michigan Emergency Medicine Residency, Muskegon, Michigan

**Keywords:** Weiss ring, posterior vitreous detachment, point-of-care ultrasound, POCUS, ocular ultrasound

## Abstract

**Case Presentation:**

A 51-year-old woman presented to the emergency department with blurred vision and a large, dark floater in her right eye. Point-of-care ultrasound (POCUS) revealed a Weiss ring, strongly suggesting a diagnosis of a posterior vitreous detachment.

**Discussion:**

Identifying a Weiss ring with POCUS may be a reasonable and easier alternative for emergency clinicians when used in conjunction with a slit-lamp examination and may aid in timely ophthalmologic management of posterior vitreous detachments.

## CASE PRESENTATION

A 51-year-old woman presented complaining of blurred vision with a large, dark floater in her right eye. Examination demonstrated a slight decrease in visual acuity of the right eye. No pupillary abnormalities or ocular dysmotility was observed. There was no evidence of trauma or foreign body on fluorescein stain, and intraocular pressures were normal via tonometer. Laboratory tests and head computed tomography demonstrated no acute abnormalities. Point-of-care ocular ultrasound revealed the diagnosis (Image 1), which was later confirmed via fundoscopic examination with ophthalmology (Image 2).^1^

## DISCUSSION

We used point-of-care ultrasound (POCUS) to identify a Weiss ring (Video). This anatomic finding is formed when peripapillary glial tissue is torn from the optic nerve head and remains adherent to the posterior vitreous cortex.^2^ The presence of a Weiss ring, when combined with a clinical presentation of sudden onset blurred vision with a floater, strongly suggests a diagnosis of a posterior vitreous detachment. The gold-standard for diagnosing posterior vitreous detachment is via slit-lamp biomicroscopy performed by ophthalmology at same-day visit.^3^ Ocular POCUS demonstrates high specificity but poor sensitivity for posterior vitreous detachment.

In the largest prospective emergency department study, POCUS identified posterior vitreous detachment with a sensitivity of 42.5% and specificity of 96.0%, indicating that positive sonographic findings are highly suggestive of disease, whereas negative findings do not reliably exclude it. Although the presence of a Weiss ring may be considered a characteristic finding of posterior vitreous detachment, published data regarding the sensitivity and specificity of ultrasound for Weiss ring detection are currently lacking. When this finding is present, however, POCUS may be a reasonable and easier alternative for emergency clinicians when used in conjunction with slit-lamp examination and may aid in timely ophthalmologic management.

*CPC-EM Capsule*

What do we already know about this clinical entity?

*Posterior vitreous detachment is a common cause of flashes/floaters and may signal a vision-threatening retinal tear.*

What is the major impact of the image(s)?

*The images show a less commonly visualized variant of a posterior vitreous detachment known as a Weiss ring, which may be misconstrued as an artifact.*

How might this improve emergency medicine practice?

*Improved identification of this variant can expedite diagnosis and lead to proper follow-up and care for a posterior vitreous detachment.*

## Supplementary Information

Video. Point-of-care ocular ultrasound recording aided in the diagnosis of a posterior vitreous detachment. Fanning back-and-forth shows the complete Weiss ring structure (arrow).

## Figures and Tables

**Image 1:**
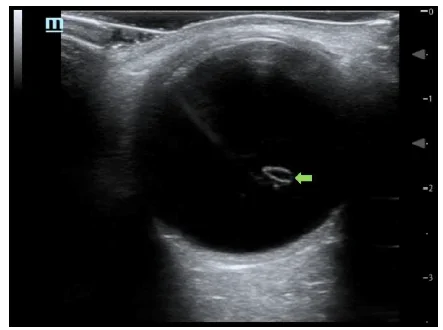
Point-of-care ocular ultrasound demonstrates a hyperechoic annular structure, known as a Weiss ring, (arrow), which is highly suggestive of a posterior vitreous detachment.

**Image 2:**
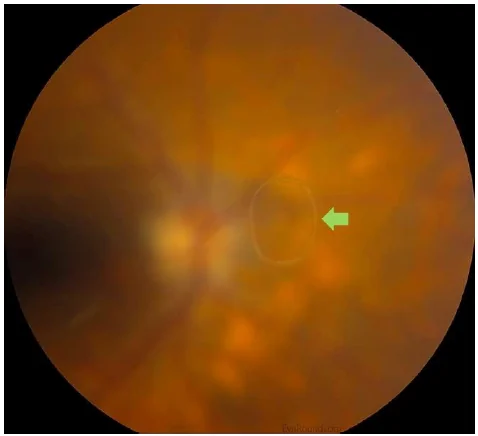
Fundoscopic examination confirmed the presence of a Weiss ring, a ring-like opacity adjacent to the optic disc (arrow).
